# The role of socioeconomic status in different trajectories of depressive symptoms in Chinese college freshmen

**DOI:** 10.3389/fpsyg.2022.945959

**Published:** 2022-08-12

**Authors:** Qingying Liu, Junying Tan, Zhengzhi Feng, Shen Tu

**Affiliations:** ^1^Department of Medical Psychology, Army Medical University, Chongqing, China; ^2^College of Public Management of GUFE, Guizhou University of Finance and Economics, Guiyang, China

**Keywords:** family socioeconomic status, subjective socioeconomic status, trajectory of depressive symptoms, depression, college freshmen

## Abstract

The associations between socioeconomic status (SES) and depressive symptoms have been found in previous studies. However, the role of SES in different trajectories of depressive symptoms in Chinese college freshmen has not been discovered. The present study aims to identify how depressive symptom trajectories are related to SES during the first semester of freshman. Six hundred fifty-two Chinese college freshmen (64.9% female) were followed 4 times across 4 months. The Latent Growth Mixture Model (LGMM) was used to identify trajectories of depressive symptoms. Multinomial Logical Regression was used to identify the influence of family socioeconomic status (FSES), subjective socioeconomic status (SSS), and demographic variables on trajectories of depressive symptoms for freshmen. Results found that college freshmen’s depressive symptoms gradually decreased during the four tests, *F*(2.758, 1795.383) = 52.642, *p* < 0.001, and there are three trajectories of depressive symptoms: normal group (Class 1, 73.1%), depression risk group (Class 2, 20.7%), and depression deterioration group (Class 3, 6.1%). The decline of SSS predicted increasing depressive symptoms. Age and left-behind experience have significant effects on trajectories of depressive symptoms. FSES, birthplace, and gender had no significant impact on trajectories of depressive symptoms. These results demonstrated that low SSS, age, and left-behind might be risk factors for the development of depressive symptoms.

## Introduction

Depression is a common mental disorder (O’Hara and Swain, 1996; Fisher et al., 2012; Friedrich, 2017) that contributes to disability (Whiteford et al., 2013) and behavior problems (Rahman et al., 2013). The prevalence of depression symptoms for college freshmen is up to 23.6% in China (Huai et al., 2013). On the one hand, Freshmen face the adaptation to new environments. Changes in interpersonal relationships, learning style, lifestyle style, and less social support from their father and mother may increase the incidence of depression and depressive symptoms (Adlaf et al., 2001; Bayram and Bilgel, 2008; Eaton et al., 2008; Chaiton et al., 2009; Rivera et al., 2013). On the other hand, stress lead to an increase in depression. Freshmen should think about their future life and career development, and begin to carry multiple pressures from their family and society as adults (Dieringer, 2012). Consequently, Freshmen’s depressive symptoms have drawn researchers’ attention.

At present, most clinicians evaluate and diagnose depression based on the critical value of the depression scale, combined with the course of the disease and personal experience. Depression is not only a category of “existence” and “nothing,” but there may also be differences in trajectories of depressive symptoms between individuals. The depressive symptoms in adolescents or early adulthood were divided into 3–5 categories using Latent Class Analysis (LCA), and Latent Growth Mixture Model (LGMM) (Needham, 2008; Rawana and Morgan, 2014; Schubert et al., 2017). However, researchers have focused more on the overall trend of depressive symptoms in a group, and less on individual differences within the same subcategory, namely group heterogeneity. The LGMM has assumed that a category has similar but not identical growth trajectories that allow variance and co-variance to vary in individuals within the same latent category, and limits cross-category equivalence. This advantage accurately distinguishes the heterogeneity between different individuals and the cause of the differences, providing a reference for the identification and intervention. Therefore, the present study investigate the trajectory of freshman’s depressive symptoms using the LGMM.

Socioeconomic status (SES) contains many disciplines, such as society, economy, psychology, and so on, including family socioeconomic status (FSES) and subjective socioeconomic status (SSS). FSES reflects one’s parents’ position in society (Bradley and Corwyn, 2002), while SSS embodies how the individual perceives and identifies his position in society (Wu et al., 2021). Previous studies have reported a close relationship between SES and mental health problems, especially depression (Brown et al., 2007; Costello et al., 2008; Ferro et al., 2015; Barr, 2018). Individuals who have a lower position and weak rights in groups will try to suppress their true feelings of things, such as anger, and restrain their behavioral responses (Tiedens et al., 2000; Frank et al., 2003) to reduce the likelihood of attachment and punishment by high social status groups. When the SSS is lower, individuals choose to suppress their negative emotional expression to avoid conflict, resulting in an increase in depression (Langner et al., 2012). Therefore, Socioeconomic status may play an important role in one’s depressive symptoms and affect the trajectory of depressive symptoms in freshmen.

Demographic variables such as gender, age, left-behind experience, and birthplace had an impact on depression. Gender and age studies from early adulthood to young adulthood were inconsistent (Galambos et al., 2006; Hong-hui and You-guo, 2013; Stapinski et al., 2013). Studies have explored that female depression is at higher levels than that of males, depression of adolescent boys is steadily decreasing with age, adolescent girls start to grow significantly at 12, peaking at 17 (Edwards et al., 2014), and the sharp decline in the age of 17–25 (Stapinski et al., 2013). But the results of gender differences are not consistent in China (Hong-hui and You-guo, 2013; Huai et al., 2013; Zhi-hua et al., 2018; Shi et al., 2021). College students with left-behind experience may bear more rejection, criticism, deprivation of love, or doting, causing an increase in depression (Hongxia et al., 2019). The proportion of depression among rural college students was higher than that among urban college students (Zhaoxia et al., 2008). However, most previous studies have examined depression from a horizontal perspective rather than a longitudinal viewpoint, so it is impossible to determine whether gender, left-behind, birthplace, and age affect the trajectory of depressive symptoms in the individuals.

The present study aims to examine the trajectory of depressive symptoms in freshmen and how depressive symptoms trajectories are related to FSES, SSS, and demographic variables. In the present study, our first hypothesis is that there are three or four trajectories of depressive symptoms in freshmen. Most freshmen belong to a stable and low-level group, but instability, risk, and the increasing group would be evident for 10–30% of the total sample. Our second hypothesis is that the trajectories of depressive symptoms will be affected by socioeconomic status in freshmen. The individual’s depressive symptoms are lower and more stable with higher FSES. On the contrary, the individual’s depressive symptoms are high and less stable with lower SSS. Our third hypothesis is that gender, birthplace, age, and left-behind effect on different trajectories of depressive symptoms are significant in freshmen.

## Materials and methods

### Participants and procedure

The present study was approved by the medical ethics committee of the army medical university. The cluster longitudinal sample data for this research were collected from 1 randomly selected college in China. After participants signed the consent form, the initial survey occurred during the third week (T1), and the follow-up waves of assessment occurred during the seventh week (T2), the eleventh week (T3), and the fifteenth weeks (T4). 680 students completed the questionnaires with guidance provided by trained researchers. 28 subjects lost due to leaving, absence, or suspension, and the remaining 652 participants (*M*age = 18.6, *SD* = 1.90) have completed a total of four tests (participation rate of 95.9%; 229 were boys, 35.1%, 423 girls, 64.9%; 99 were born in the urban, 15.2%, 253 were born in the rural, 84.8%; 354 were left-behind students, 54.4%, 297 were not left-behind students, 45.6%). There was no significant difference in the results of the bilateral *T*-test in the first measurement of the subjects with loss and continuous participation, *t*(678) = 0.239, *p* = 0.811, indicating that there was no structured loss of the sample. SES is a relatively stable variable due to the short tracking time (only 4-month), so family SES was measured only at T1, and SSS was measured at T2. Depression symptoms were unstable and measured at each time point.

### Measures

#### Depressive symptoms

Depressive symptoms were measured using Beck Depression Rating Scale (BDI). The BDI is a 21-item measure of “symptoms-Attitude” for individuals. The 21-item scored 0–3 (Beck, 1961; Zhen et al., 2011; Shui-Lin, 2019). Total scores (the sum of 21 questions) represent the severity of depressive symptoms. In this study, Cronbach’s alpha coefficients of the BDI at four measurement times were 0.837, 0.872, 0.906, and 0.922, respectively.

#### Family Socioeconomic Status

Family SES contained home possessions, parental educational level, and parental occupational prestige and was measured using the Organization for Economic Cooperation and Development’s (OECD, 2012) Program for International Student Assessment method. The calculation methods of family SES were provided by Cheng and Wu (Cheng et al., 2015; Wu et al., 2021). In present study, the range of family SES was −2.19 to 3.06.

#### Subjective Socioeconomic Status

Subjective Socioeconomic Status was measured by the Chinese version of the Subjective Socioeconomic Status Scale (SSS), which was translated by Yu-Ning et al. (2014) from Adler’s scale (MacArthur Scale of Subjective Social Status). The scale contains two items. Item 1 (social status) was associated with traditional SES indicators in which individuals assess their position in the overall social environment. Item 2 (Community status) was associated with a living environment in which individuals evaluate their location in the community. Each item scored 1–10. The Cronbach’s alpha coefficients of two items were 0.649. Lower scores reflected lower SSS.

### Data analyses

Data analysis contains the following three steps. (1) Preliminary analyses included descriptive statistics, correlation analysis, and one-way repeat-measure ANOVA. (2) Latent Growth mixture modeling (LGMM) identifies classes of trajectories of depressive symptoms in freshmen using Mplus 7.4. Standard criteria for determining the number of classes included the following factors: the Bayesian Information Criteria (BIC), the Sample-Size Adjusted Bayesian information criterion (aBIC), the Lo-Mendel-Rubin likelihood ratio test (LMRT), the bootstrapped likelihood ratio test (BLRT), Entropy provided a degree of separation between classes (Entropy). According to the principle of higher Entropy, lower AIC, BIC, aBIC, LMR, and BLRT (*p* < 0.05), the model fitted better (Meng-Cheng et al., 2017). (3) The Multiple Logic Regression Analysis examines the predictors of depression symptoms. The predictors included the latent class (as a dependent variable), Gender, birthplace, and left-behind (as independent variables), Family socioeconomic status (Family SES), Subjective socioeconomic status of adults (SSS), and age (as covariates).

## Results

### Control and test of common method bias

First, the present study controlled the data collection process according to the suggestions of the relevant research (Gang and Dajun, 2018). The depression was measured by a 4-point scoring method. The family SES is converted by demographic variables. The SSS is measured by a graphical 10-point scoring. Second, common method bias was tested by using the Harman factor test. According to test family SES, SSS, and depression, the results showed that eigenvalues of 21 factors are higher than 1, where the factor with the largest eigenvalue explained 23.97% of the variance variation, below the 40% critical criterion. In a word, the common method bias was not severe and did not significantly interfere with the results in the present study.

### Preliminary analyses

Firstly, as shown in Table 1, the depressive symptoms of freshmen in four-time points were significantly positively correlated (*p* < 0.01). One-way repeat-measure ANOVA was used to perform whether there were differences among four test scores of depression symptoms in freshmen. (1) Mauchly’s Test of Sphericity results showed a significant difference, χ^2^ = 89.33, *p* < 0.001, epsilon(ϵ) = 0.919), indicating not satisfying the spherical hypothesis condition. Huynh-Feldt correction results showed a significant difference, *F*(2.758, 1795.383) = 52.642, *p* < 0.001, Parietal Eta Square = 0.075, ω^2^ = 0.056. Pairwise comparison of four BDI tests showed that: the first time scores of BDI were higher than the third (*p* < 0.05) and the fourth Tests (*p* < 0.05); the second Test BDI score was higher than the third (*p* < 0.05) and the fourth Tests (*p* < 0.05); the third Test BDI score was higher than the fourth time (*p* < 0.05), indicating that freshmen’s depressive symptoms gradually decreased during the four tests. Secondly, SSS and the depressive symptoms of the four-time points were significantly relevant, *r* = −0.226–0.245, *p* < 0.01, 95% CI (−0.317, −0.135). Thirdly, the depressive symptoms exhibited skewness and kurtosis above 1, indicating a non-normal distribution. Therefore, Robust Maximum Likelihood Estimator (MLR) method was used when Latent Growth mixture modeling performed.

**TABLE 1:** Descriptive statistics and correlations among the study variables.

	1	2	3	4	5	6
1. T1BDI	1					
2. T2BDI	0.744**	1				
3. T3BDI	0.615**	0.733**	1			
4. T4BDI	0.588**	0.679**	0.723**	1		
5. SSS	−0.238**	−0.226**	−0.245**	−0.229**	1	
6. Family SES	–0.047	–0.049	–0.032	–0.02	0.248**	1
M	8.535	8.135	7.074	5.983	10.104	0.003
SD	6.861	7.084	7.341	7.269	2.578	0.999
Skewness	1.191	1.477	1.362	1.552	0.106	0.678
Kurtosis	1.399	2.901	1.556	2.104	1.072	0.325

*n* = 652, **p < 0.01, T, times; SSS, subjective socioeconomic status; Family SES, family socioeconomic status.

### Trajectories of depressive symptoms in freshmen

**FIGURE 1:**
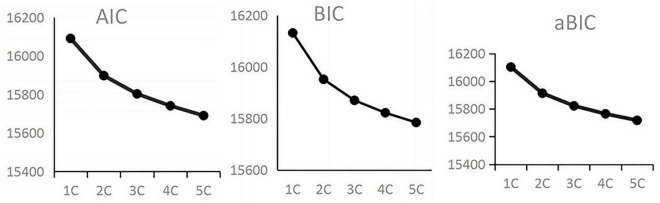
AIC, BIC, and aBIC in different class of latent growth mixture model (LGMM model).

A Latent Growth Mixture Modeling (LGMM) of 1–5 latent classes was established in the present study. The better fitting indices were shown in Table 2. From Table 2, the log (L), AIC, BIC, aBIC information index gradually decreased with the increase of model class, so it was difficult to determine the best fitting model only according to these information indices. Grabble diagram of AIC, BIC, and aBIC should be observed to select the best model (Petras and Masyn, 2010). The grabble diagram of AIC, BIC, and aBIC in different latent class models was shown in Figure 1. From Figure 1, AIC, BIC, and aBIC all showed obvious inflection points at 2C and began to drop slowly at 3C, which indicated that 2 or 3 latent class models should be appropriate. Entropy values gradually increased from 2C to 5C, and were higher than 0.84, indicating that the classification’s accuracy was more than 90% (Meng-Cheng et al., 2017). The *p*-value of LMR and BLRT for 2 and 3 latent class models was < 0.05. The *p*-value of the LMR of 4 and 5 latent class models was > 0.05, which indicated that 3 latent class models were significantly better than 2, 4, and 5 latent class models. From the class probability in the Table, the Class 3 model refined the distinction of subjects. Therefore, considering the above indicators, 3 trajectories of depressive symptoms were more reasonable.

**TABLE 2:** Comparisons of latent growth mixture modeling for BDI.

Model	K	log(L)	AIC	BIC	aBIC	Entropy	LMR	BLRT	Class probability
1C	9	−8036.556	16091.112	16131.433	16102.858				
2C	12	−7936.971	15897.941	15951.702	15913.602	0.887	0.0137	0	0.151/0.849
3C	15	−7886.543	15803.085	15870.286	15822.661	0.897	0.0316	0	0.061/0.207/0.73.1
4C	18	−7852.713	15741.426	15822.066	15764.916	0.914	0.0597	0	0.018/0.22/0.67/0.09
5C	21	−7824.231	15690.461	15784.542	15717.867	0.923	0.7316	0	0.006/0.664/0.018/ 0.219/0.092

K, Number of Free Parameters. AIC, Akaike information criterion; BIC, Bayesian information criterion; aBIC, Sample-Size Adjusted BIC; LMR, Lo-Menell-Rubin Adjust LRT Test; BLRT, bootstrapped likelihood ratio test.

From average latent class probabilities for most likely latent class membership (Row) by latent class (column) of Table 3, the membership (row) attribution of each latent class was from 88.7 to 97.2% (column), indicating that the results of 3 trajectories of depressive symptoms were more credible.

**TABLE 3:** Average latent class probabilities for most likely latent class membership (Row) by Latent Class (Column).

	Class 1 (%)	Class 2 (%)	Class 3 (%)
Class 1	97.2	2.8	0
Class 2	8.2	88.7	3.2
Class 3	0	4.8	95.2

### The characteristics in relation to development trajectory of depressive symptoms in freshmen

**FIGURE 2:**
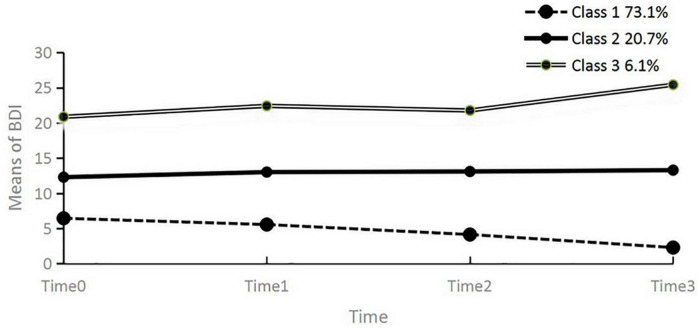
Latent class growth trajectory map of LGMM (*k* = 3).

Based on the development trajectory of depressive symptoms in freshmen, the characteristics of three latent classes were examined. The 3 latent class indices and growth trajectory maps were presented in Table 4 and Figure 2. The mean intercept of three latent classes were: Class 1 6.664 (*p* < 0.01), Class 2 12.307 (*p* < 0.01), Class 3 20.156 (*p* < 0.01), suggesting a significant individual difference (lower Class 1, higher Class 2 and Class 3) in the initial values of depression symptoms. The mean slope of 3 latent classes was Class 1 −1.418 (*p* < 0.01), Class 2 0.233 (*p* > 0.05), and Class 3 1.502 (*p* < 0.01), suggesting a significant change concerning the levels of depression symptoms in Class 1 and Class 3 during the time of four BDI test. This result indicated the depressive symptoms in Class 1 decreased, Class 3 increased significantly, and the rate of change in Class 1 was lower than in Class 3. Combined with mean values of intercept and slope, the characteristics of 3 latent classes were as follows: the initial score of Class 1 was lower, and the rate of decline was faster, accounting for 73.1% of the population, which could be named “normal group”; The initial score of Class 2 was higher, the rate of change was not obviously in four measurements, and the depressive symptoms always maintained medium levels (10–15 points) during four BDI test, accounting for 20.7% of the population, which could be named “depression risk group”; The initial score of Class 3 was very high, and depressive symptoms increased slowly in four measurements, accounting for 6.1% of the population, which could be named “depression deterioration group.”

**TABLE 4:** Means of intercept and slope from the latent growth mixture model of BDI.

Class		Estimate	S.E.	Est./S.E.	*P*-value
Class 1	Intercept(I)	6.664	0.262	25.483	0.000
	Slope(S)	−1.418	0.089	−15.881	0.000
Class 2	Intercept(I)	12.307	0.812	15.154	0.000
	Slope(S)	0.233	0.234	0.998	0.318
Class 3	Intercept(I)	20.156	1.801	11.191	0.000
	Slope(S)	1.502	0.660	2.276	0.023

### Multinomial logistic regression analysis: The influence of FSES, subjective socioeconomic status and demographic variables on trajectories of depressive symptoms in freshmen

To investigate the influence factors of the trajectory of depressive symptoms in freshmen, the present adopted the method of Multinomial Logistic Regression Analysis. The trajectory of depressive symptoms (3C) was taken as a dependent variable, gender (male = 1, female = 0), left-behind (left = 1, no-left = 0), and birthplace (urban = 1, rural = 0) as independent variables, FSES, SSS, and age as covariates in the regression model. The results were shown in Table 5.

**TABLE 5:** The results of multinomial logistic regression.

Variables	Depression deterioration group vs. normal group				Depression risk group vs. normal group				Likelihood ratio detection	
	B	Wald	Exp(B)	95% CI	B	Wald	Exp(B)	95% CI	−2LL	X^2^
Family SES	0.23	1.40	1.26	(0.86,1.83)	0.08	0.46	1.08	(0.86,1.36)	876.70	1.65
SSS	–0.25	11.61**	0.78	(0.68,0.90)	–0.27	34.91**	0.77	(0.70,0.84)	920.34	45.29**
Gender	–0.16	0.20	0.68	(0.44,1.67)	0.26	1.45	1.30	(0.85,1.99)	876.96	1.90
Age	–0.15	7.61**	0.86	(0.78,0.96)	–0.01	0.03	0.99	(0.67,1.13)	881.56	6.51**
Birthplace	0.11	0.05	1.12	(0.42,2.96)	0.64	5.41**	1.90	(1.11,3.27)	880.26	5.21
Left-behind	0.67	3.34	1.99	(0.96,4.13)	0.59	7.32**	1.81	(1.18,2.78)	884.76	9.71**

**p < 0.01. Normal group as reference group.

Subjective socioeconomic status had a significant predictive effect on the trajectory of depressive symptoms. For the normal group vs. depression deterioration group, subjective social status was found to significantly decrease the probability of freshmen belonging to the depression deterioration group than the normal group, OR = 0.78, *p* < 0.01, 95% CI (0.68, 0.90). For the normal group vs. the depression risk group, subjective social status decreased the likelihood of belonging to the depression risk group than the normal group, OR = 0.77, *p* < 0.01, 95% CI (0.70, 0.84). In a word, the results showed that individuals with high subjective social status were less likely to fall into depression.

Age had a significant predictive effect on the trajectory of depressive symptoms. For the normal group vs. depression deterioration group, the present study found age significantly decreases the probability of belonging to the depression deterioration group, OR = 0.86, *p* < 0.01, 95% CI (0.78, 0.96). Compared to the normal group, individuals were 1.81 times more likely to be included in the depression-risk group with each additional unit of the left-behind experience, OR = 1.81, *p* < 0.01, 95% CI (1.18, 2.78). The likelihood ratio detection of birthplace was not significant (*p* > 0.05), so the effect of birthplace on the trajectory of depressive symptoms did not discuss in the present study. FSES and gender had no significant predictive effect on the trajectory of depressive symptoms in freshmen (*p* > 0.05).

## Discussion

The present study aimed to examine the trajectory of depressive symptoms by using the LGMM and the influence factors adopted the method of Multinomial Logistic Regression Analysis in freshmen during a 4-month follow-up survey of BDI. Therefore, the present study analyzed and discussed characteristics related to the trajectory of depressive symptoms in freshmen, the effect of SSS, FSES, and demographic variables on the trajectory of depressive symptoms.

### The features of trajectory of depressive symptoms in freshmen

Freshmen’s depressive symptoms gradually decreased during the four tests from one-way repeat-measure ANOVA, which indicated that a high level of depression in freshmen might be a manifestation of stress in high-middle school or a stress response during the transition period after enrollment (Gang et al., 2016). However, the present study showed significant group heterogeneity in freshmen. Based on the results of LGMM, the mean of intercept, and slope, the trajectory of depressive symptoms was divided into three different latent classes in freshmen. The majority (73.1%) maintained a low level of depressive symptoms, decreased trend during 4-months, and were named the “normal group.” The results demonstrated that many freshmen’s depression symptoms might decrease with longer school time, environmental adaptation, and interpersonal relationship improvement (Schubert et al., 2017). The scores of depressive symptoms (accounting for 20.7%) were maintained at the moderate level (10–15 points), and the slop was not significant in Class 2 during 4 BDI tests, which were named “depression risk group.” Depressive symptoms of the depression risk group were high and long-term stability and would descend or rise in the future. So the measures that should be taken are to keep continuous attention, actively carry out some interventions, and maintain positive psychological attention to them to decrease their depressive symptoms. The initial scores of depressive symptoms in Class 3 (accounting for 6.1% of the population) presented a high level and increased trend during four BDI measurements, which was named the “depression deterioration group.” This population was likely to develop into “depression patients.” What should be done is to examine their susceptibility factors, such as personality characteristics, stress events, and negative cognitive processing, and carry out direct intervention and continuous tracking to reduce their levels of depression.

### Effect of socioeconomic status on trajectory of depressive symptoms in freshmen

The present study found that FSES had no significant impact on the trajectory of depressive symptoms in freshmen, which was not consistent with previous studies (Brown et al., 2007; Bayram and Bilgel, 2008; Costello et al., 2008; Ferro et al., 2015). While SSS had a significant impact on the trajectory in freshmen, which was similar to previous studies (Gang et al., 2016). Previous studies suggested that lower family financial income, lower academic levels, and lower social status with careers could increase depression (Brown et al., 2007; Costello et al., 2008; Ferro et al., 2015). However, our results were inconsistent with these studies. Individuals with lower SSS were inclined to belong to the “depression risk group” and “depression deterioration group,” suggesting that the state of depression increased significantly with progressively lower SSS. In addition to academic performance, college freshmen would attend to their family’s economic situation, external dressing, even parental careers, and other aspects. According to emotion suppression theory, individuals with lower FSES may control their behavior, and compare themself with others in negative processing perspectives, which may accompany a decline in SSS and an increase in depression (Langner et al., 2012).

The present study did not find a significant effect of family SES on depressive symptoms. However, this effect of family SES on depressive symptoms may be caused by decreasing SSS. College students with low family SES may face various threats or challenges in life and study, experience more negative emotions, are prone to form negative cognition and evaluation, and produce a low SSS (Xiaoshan et al., 2022). Individuals with low SSS may excessively adopt the emotional dysregulation strategies accompanied by strong negative emotions to deal with various stresses or challenges in life, which increases their susceptibility to depression (Wenyan et al., 2021). Therefore, improving individuals’ SSS may effectively reduce the level of depression when family SES is temporarily difficult to change. To objectively view family SES and actively improve SSS, making individuals adopt healthy regulation strategies and optimistic cognitive evaluation methods can effectively reduce the depression status of freshmen.

### Demographic variables effect on trajectory of depressive symptoms in freshmen

The analysis of demographic variables which affected the trajectory of depressive symptoms among freshmen found that: (1) older individuals were less likely to be included in the depression deterioration group than younger individuals. This result is similar to previous studies (Radloff, 1991; Hankin et al., 1998; Jinqin and Zhiyan, 2016). The first semester of college is the transition period from late adolescence to early adulthood. Freshmen face many stresses, such as separating from their parents, conducting career planning, and establishing friendly interpersonal and intimate relationships. Many individuals can gradually resolve these transition stresses, resulting in a low level of depression. (2) Freshmen with left-behind experience are more likely to be included in the depression risk group. This result is consistent with many studies in China (Zhaoxia et al., 2008; Hongxia et al., 2019). College students with left-behind experience may experience more neglect, criticism, or doting during their childhood, and face more pressure during the transition period, resulting in a relatively high level of depression. (3) The present study found no significant gender effect on the trajectory of depressive symptoms in freshmen, which was consistent with previous studies in China (Hong-hui and You-guo, 2013; Gang et al., 2016; Ai Lou and Xian Min, 2020), and inconsistent with other countries (Stapinski et al., 2013; Schubert et al., 2017; Lewis et al., 2020). On the one hand, many studies found no gender differences using the SDS or BDI scale, and rarely considered the duration of depressive symptoms. Therefore, the participants were depression-susceptible individuals in normal individuals rather than clinical patients. On the other hand, cross-cultural education differences existed in different countries. In China, college girls can get away from the original “male preference for women” and get unanimous admiration and respect from family and friends, and better development opportunities in the future. Therefore, female college freshmen’s depression state decreases significantly in college, and there is no significant gender effect.

### Limitations and future studies

The present study examined the trajectory of depressive symptoms in freshmen using LGMM through 4-month tracking. We found three latent classes in freshmen, and a decline in SSS made individuals more inclined to the “depression risk group” and the “depression deterioration group.” But there are some improved areas in this study: (1) As the present study tracked only students in one college school at the intensive interval, the conclusions could not extend to the national freshmen during the entire college period. Future studies should extend the sampling range and the interval period. (2) The tracking samples were only college freshmen and lack of peers who are not admitted to college as a control group. Therefore, the increase in freshmen’s depression may be a general adaptation caused re-understanding of socioeconomic status or a side effect of freshmen actively adapting to the new university environment. Future studies should consider extending unenrolled peers as a control group and comparing the depression differences between freshmen and unenrolled peers. (3) The present study did not explore the effects of protective and susceptible factors, such as social support, positive attribution, harmonious parent-child relationship, and over-generalization. Future research should combine a comprehensive and systematic study of protective factors and susceptibility factors to reveal the influence factors on a trajectory of depressive symptoms. (4) The SES of the present study was measured once and not tracked. Considering the short duration of this longitudinal study, the changes in SES may be modest. However, college students may also change their SSS during adapting to the new environment. Therefore, SES should be measured repeatedly to examine the relationship between SES changes and depression. (5) The SES was measured using self-report measures. This method is time-consuming, labor-intensive, and not suitable for large-scale surveys. With the development of big data technology and machine learning, future research should automatically evaluate their social-economic status by integrating multi-dimensional data and various algorithms.

## Conclusion

In sum, the present study found three trajectories of depressive symptoms in freshmen: normal group, depression risk group, and depression deterioration group. The decline in the SSS of freshmen means that individuals are more likely to be in the depression risk group and depression deterioration group, specifically. In brief, a low level of SSS means a high level of depressive symptoms. Age and left-behind rather than family SES, birthplace, and gender have a significant impact on the trajectory of depressive symptoms in freshmen. Future research would benefit from extending the sampling range and interval period, examining the protective and risk factors of depressive symptoms, as well as tracking the SES in big data technology and machine learning.

## Data availability statement

The raw data supporting the conclusions of this article will be made available by the authors, without undue reservation.

## Ethics statement

The studies involving human participants were reviewed and approved by the Ethics Committee of the Army Medical University. The patients/participants provided their written informed consent to participate in this study.

## Author contributions

All authors listed have made a substantial, direct, and intellectual contribution to the work, and approved it for publication.
